# Design of Partial Mueller-Matrix Polarimeters for Application-Specific Sensors

**DOI:** 10.3390/s25196249

**Published:** 2025-10-09

**Authors:** Brian G. Hoover, Martha Y. Takane

**Affiliations:** 1 Advanced Optical Technologies, Inc., Albuquerque, NM 87123, USA; 2 Instituto de Matemáticas, Universidad Nacional Autónoma de México, Ciudad de México 04510, Mexico; takane@im.unam.mx

**Keywords:** Mueller matrix, Stokes vector, remote sensing, laser polarimeter, vector space, cone theory, subspace projection

## Abstract

At a particular frequency, most materials and objects of interest exhibit a polarization signature, or Mueller matrix, of limited dimensionality, with many matrix elements either negligibly small or redundant due to symmetry. Robust design of a polarization sensor for a particular material or object of interest, or for an application with a limited set of materials or objects, will adapt to the signature subspace, as well as the available modulators, in order to avoid unnecessary measurements and hardware and their associated budgets, errors, and artifacts. At the same time, measured polarization features should be expressed in the Stokes–Mueller basis to allow use of known phenomenology for data interpretation and processing as well as instrument calibration and troubleshooting. This approach to partial Mueller-matrix polarimeter (pMMP) design begins by defining a vector space of reduced Mueller matrices and an instrument vector representing the polarization modulators and other components of the sensor. The reduced-Mueller vector space is proven to be identical to $R^{15}$ and to provide a completely linear description constrained to the Mueller cone. The reduced irradiance, the inner product of the reduced instrument and target vectors, is then applied to construct classifiers and tune modulator parameters, for instance to maximize representation of a specific target in a fixed number of measured channels. This design method eliminates the use of pseudo-inverses and reveals the optimal channel compositions to capture a particular signature feature, or a limited set of features, under given hardware constraints. Examples are given for common optical division-of-amplitude (DoA) 2-channel passive and serial/DoT-DoA 4-channel active polarimeters with rotating crystal modulators for classification of targets with diattenuation and depolarization characteristics.

## 1. Introduction

Techniques in active or laser polarimetry are gaining popularity for applications in remote sensing [1,2], microscopy [3], biomedical optics [4,5], nanostructure characterization [6,7], and industrial process control [8], among others [9]. The most general active polarimeters, also known as *Mueller-matrix polarimeters* (MMPs), measure the complete 4×4 Mueller matrix, which may be considered the complete *polarization signature* of the illuminated object, by recording the intensity or irradiance of polarized light reflected, transmitted, or scattered by the object for a minimum of 16 polarimeter channels, which are combinations of transmitter and receiver polarization states determined by modulator settings [10,11]. Techniques that utilize MMPs are in the fields of *Mueller-matrix polarimetry* [12,13] or *Mueller-matrix ellipsometry* [14]. The evolution of active and laser polarimetry from laboratory to field instruments has been slow due mainly to limitations on speed and miniaturization imposed by the need to make 16 measurements per signature. While laboratory polarimeters should be capable of measuring general 16-dimensional polarization signatures [15], such generality is not needed in field instruments designed to sense specific targets or conditions in specific scenarios. Laboratory databases of optical polarization signatures reveal that classification algorithms can be trained on subsets of the Mueller matrix [16,17,18,19]; it is therefore practical to design faster, smaller active polarimeters that measure only those characteristic subsets, ie, *partial Mueller-matrix polarimeters* (pMMPs) [20,21].

Prior published approaches for pMMP design suffer from over-simplifying assumptions and/or computational difficulties. The DRM approach of Tyo et al. [20,21] applies vector algebra to the set of Mueller matrices, which however do not form a vector space, and relies on the instrument-matrix pseudoinverse, which is prone to computational instability without proper conditioning [22]. The approach of Anna et al. [23] makes a priori simplifications of the scene space, particularly that certain Mueller-matrix elements can be neglected in the measured data, which could break down and corrupt the results if the polarimeter encounters unanticipated objects. The technique introduced here, by operating on the vector space of *reduced* Mueller matrices $M^{\prime }$, defined in Section 2 and proven identical to $R^{15}$ in Appendix B, makes no simplifying assumptions about the measured scene data, admitting any Mueller matrix into scene space. A priori signatures are used to simplify the *target* Mueller matrices the pMMP is designed to measure, and the reduced irradiance is expressed as the inner product of the reduced target matrix and the reduced instrument matrix, which are vectors in $M^{\prime }$. The method thereby avoids use of a pseudoinverse, instead expressing and maximizing the reduced irradiance as a standard inner product on the reduced-Mueller space $M^{\prime }$. A priori signatures are used to simplify the target Mueller matrices, but no assumptions are made about non-target components in the scene, and pseudoinverse is avoided.

The new pMMP design method uniquely optimizes over channel and signature combinations. In addition to any linear combination of channel irradiances, which may be casually referred to as *linear-combination channels* (see Section 3.1), the technique can combine different signatures into pMMP channel design. In imaging it may be assumed that the different signatures, to which correspond specific objects or materials, are spatially resolved, although this is not essential. The new technique can uniquely train a pMMP channel, or group of channels, to one signature, and train a second channel or group to another signature, et cetera. While at this stage it does not consider the effects of instrument noise, and so assumes the signal-to-noise ratio (SNR) is sufficiently high, it naturally optimizes according to scene contrast or *signal-to-background ratio* (SBR), shown in Section 4 using a *nonlinear* channel combination. Section 4 also shows how the new method can optimize a linear combination of measured independent channels to lower the classifier dimension and variance to the limit of a maximally biased homogeneous classifier for a particular target matrix as a linear combination of all available channels. Also in Section 4, potential classification algorithms based on the new pMMP design method are compared with other classifiers that have utilized Mueller matrices. The appendices review relevant aspects of the Mueller cone, leading to proof of the reduced-Mueller vector space.

## 2. Reduced Mueller-Matrix Vector Space

The *Stokes parameters* $S={\left[\begin{array}{cccc} S_{0} & S_{1} & S_{2} & S_{3} \end{array}\right]}^{T}$ quantitatively describe the polarization state of an electromagnetic wave, and the 4×4 *Mueller matrix* M is the linear transformation of the Stokes parameters upon interaction with a material object or medium [10]:

(1) $$S_{o}={\mathrm{MS}}_{i}.$$

The Mueller matrix can be considered the *polarization signature* of the object or medium in the Stokes basis. While conventional polarization mathematics invokes concepts of vector spaces—the Stokes parameters are even typically referred to as the *Stokes vector*—due to the fact that they do not contain additive inverses, neither the set of physical Stokes parameters nor the set of physical Mueller matrices form actual vector spaces. The set of Stokes “vectors”

(2) $$K=\{S\in R^{4}|\;S_{0}\geq 0\;\&\;S^{T}\mathrm{GS}\geq \;0\},$$

where G= diag$\left(1,-1,-1,-1\right),$ forms a solid cone with the usual norm of $R^{4}$. As reviewed in Appendix A, a cone *K* is a set closed under *non-negative* linear combinations within a normed real vector space, which for the Stokes parameters is $R^{4}.$ The condition $S^{T}\mathrm{GS}\geq$ 0 is equivalent to a degree of polarization 0≤P≤1. The set of Mueller matrices, which map the cone *K* into itself, also forms a cone,

(3) $$\tilde{K}=\left\{M\in R^{4\times 4}|\;M\left(K\right)\subseteq K\right\},$$

as proven in Appendix A. A cone differs fundamentally from a vector space [24], and theorems derived for vector spaces do not generally hold for Stokes vectors and Mueller matrices. In this section a vector space of reduced Mueller matrices is defined on which the theorems of vector algebra can be universally applied. This vector space is then utilized to derive channels of partial Mueller-matrix polarimeters and polarization classifiers for general configurations and example optical pMMPs.

**Figure 1:**
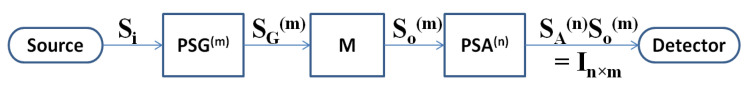
Block diagram of a general partial Mueller-matrix polarimeter (pMMP). A polarization-state generator (PSG) with *M* hardware settings combined with a polarization-state analyzer (PSA) with *N* hardware settings forms an $\left(M\times N\right)$-channel pMMP. **M** denotes the Mueller matrix of the illuminated scene.

Figure 1 is a block diagram of a general pMMP. The source electromagnetic wave, described by Stokes vector $S_{i},$ is transformed to the illumination state $S_{G}^{\left(m\right)},$ of which there can be *M* tunable settings in the *polarization-state generator* (PSG). In this paper the illumination so produced is assumed to be a plane wave or an elementary paraxial beam when it interacts with the scene/object/material, with constant polarization state over the illuminated area. Mueller matrix M represents the illuminated scene/object/material of interest. For imaging, the scene comprises a variety of objects or materials, each with different M, that are spatially resolved in the recorded image. Through reflection, transmission, and/or scattering, the scene transforms the illumination state as $S_{o}^{\left(m\right)}={\mathrm{MS}}_{G}^{\left(m\right)}.$ A portion of the reflected, transmitted, or scattered light is collected by the receiver and directed through the *polarization-state analyzer* (PSA), which can be in *N* tunable settings. As will be expanded below, the PSG and PSA are themselves represented by Mueller matrices, which are functions of their modulator settings. In particular, the PSA is taken to include the detector or camera, which, by recording only irradiance, reduces the PSA transformation to the first row of its Mueller matrix, which is denoted $S_{A}^{\left(n\right)}.$ Any measurement of any linear polarization property of any object can be expressed as a pMMP.

Adapting from Tyo et al. [20], the PSA and PSG Stokes parameters for the ${\left(m\times n\right)}^{th}$ pMMP channel are defined as

(4) $$\begin{array}{ccc} S_{G}^{\left(m\right)} & \equiv & {\left[\begin{array}{cccc} \sigma_{0}^{\left(m\right)} & \sigma_{1}^{\left(m\right)} & \sigma_{2}^{\left(m\right)} & \sigma_{3}^{\left(m\right)} \end{array}\right]}^{T}\;\mathrm{and}\;\;\; \end{array}$$

(5) $$\begin{array}{ccc} S_{A}^{\left(n\right)} & \equiv & \left[\begin{array}{cccc} s_{0}^{\left(n\right)} & s_{1}^{\left(n\right)} & s_{2}^{\left(n\right)} & s_{3}^{\left(n\right)} \end{array}\right],\;\;\;\;\; \end{array}$$

for the $m^{th}$ PSG hardware setting and the $n^{th}$ PSA hardware setting. $S_{G}$ represents the electromagnetic field exiting the PSG ($\sigma_{i}^{\left(m\right)}\equiv S_{Gi}^{\left(m\right)}$), while $S_{A}$ is the top row of the Mueller matrix representing the PSA. For an active or laser polarimeter, as in Section 3.2, $S_{G}$ and $S_{A}$ are typically on the boundary of the Stokes cone *K*, while for a passive polarimeter, as in Section 3.1, $S_{A}$ is typically on the boundary while $S_{G}$ is in the interior of K. $S_{o}$ is also in the interior of K, in either passive or active mode, if the scene M causes any depolarization. A polarimeter irradiance measurement is then expressible as

(6) $$I_{m\times n}=S_{A}^{\left(n\right)}{\mathrm{MS}}_{G}^{\left(m\right)}=\sum_{i,j=0}^{3}s_{i}^{\left(n\right)}M_{ij}\sigma_{j}^{\left(m\right)}=\sum_{i,j=0}^{3}W_{ij}^{\left(m\times n\right)}M_{ij},$$

where

(7) $$W^{\left(m\times n\right)}\equiv \left[\begin{array}{cccc} \sigma_{0}^{\left(m\right)}s_{0}^{\left(n\right)} & \sigma_{1}^{\left(m\right)}s_{0}^{\left(n\right)} & \sigma_{2}^{\left(m\right)}s_{0}^{\left(n\right)} & \sigma_{3}^{\left(m\right)}s_{0}^{\left(n\right)}\\ \sigma_{0}^{\left(m\right)}s_{1}^{\left(n\right)} & \sigma_{1}^{\left(m\right)}s_{1}^{\left(n\right)} & \sigma_{2}^{\left(m\right)}s_{1}^{\left(n\right)} & \sigma_{3}^{\left(m\right)}s_{1}^{\left(n\right)}\\ \sigma_{0}^{\left(m\right)}s_{2}^{\left(n\right)} & \sigma_{1}^{\left(m\right)}s_{2}^{\left(n\right)} & \sigma_{2}^{\left(m\right)}s_{2}^{\left(n\right)} & \sigma_{3}^{\left(m\right)}s_{2}^{\left(n\right)}\\ \sigma_{0}^{\left(m\right)}s_{3}^{\left(n\right)} & \sigma_{1}^{\left(m\right)}s_{3}^{\left(n\right)} & \sigma_{2}^{\left(m\right)}s_{3}^{\left(n\right)} & \sigma_{3}^{\left(m\right)}s_{3}^{\left(n\right)} \end{array}\right]$$

is the *dyadic product* or “outer product” of the PSA and PSG Stokes vectors: $W^{\left(m\times n\right)}\equiv S_{G}^{\left(n\right)}S_{A}^{\left(m\right)}.$ Converting from $R^{4\times 4}$ to $R^{16}$, i.e.,

$$\begin{array}{ccc} \tilde{W} & = & \left[\begin{array}{ccccccccccc} W_{0} & W_{1} & W_{2} & W_{3} & W_{4} & W_{5} & \ldots & W_{12} & W_{13} & W_{14} & W_{15} \end{array}\right]\;\;\;\; \end{array}$$

(8) $$\begin{array}{ccc} & \equiv & \left[\begin{array}{ccccccccccc} \sigma_{0}s_{0} & \sigma_{1}s_{0} & \sigma_{2}s_{0} & \sigma_{3}s_{0} & \sigma_{0}s_{1} & \sigma_{1}s_{1} & \ldots & \sigma_{0}s_{3} & \sigma_{1}s_{3} & \sigma_{2}s_{3} & \sigma_{3}s_{3}\; \end{array}\right]\\ & & \mathrm{and}\; \end{array}$$

$$\begin{array}{ccc} \tilde{M} & = & \left[\begin{array}{ccccccccccc} {\bar{M}}_{0} & {\bar{M}}_{1} & {\bar{M}}_{2} & {\bar{M}}_{3} & {\bar{M}}_{4} & {\bar{M}}_{5} & \ldots & {\bar{M}}_{12} & {\bar{M}}_{13} & {\bar{M}}_{14} & {\bar{M}}_{15} \end{array}\right]\;\;\; \end{array}$$

(9) $$\begin{array}{ccc} & \equiv & \left[\begin{array}{ccccccccccc} M_{00} & M_{01} & M_{02} & M_{03} & M_{10} & M_{11} & \ldots & M_{30} & M_{31} & M_{32} & M_{33}\; \end{array}\right] \end{array}$$

the polarimeter irradiance measurement, Equation (6), can be written as

(10) $$I_{m\times n}=\sum_{i=0}^{15}W_{i}^{\left(m\times n\right)}{\bar{M}}_{i}.$$

$\tilde{W}$ is the *instrument vector* to be optimized for pMMP design.

The dot-product of Equation (10) is over $R^{16}$, which is isomorphic to $R^{4\times 4},$ which contains, in addition to Mueller matrices, all non-physical real 4×4 matrices, including those with ${\bar{M}}_{0}<0.$ The range of pMMP solutions that could be found by inverting this equation is unconstrained, less unique, and computationally unstable. The sets of physical $\tilde{W}$ and $\tilde{M}$ do not form vector spaces since they lack additive inverses, so there is no inner product on $R^{16}$ that is constrained to conventional Mueller matrices.

A Mueller vector space and inner product can however be created by reducing the measured irradiance as follows. Unpolarized illumination of irradiance $\sigma_{0}$ is represented by $S_{G}={\left[\begin{array}{cccc} \sigma_{0} & 0 & 0 & 0 \end{array}\right]}^{T},$ while a non-polarizing PSA (e.g., a conventional non-polarizing camera) is represented by $S_{A}=\left[\begin{array}{cccc} s_{0} & 0 & 0 & 0 \end{array}\right].$ The corresponding *unpolarized* irradiance is

(11) $$I_{U}=\sigma_{0}s_{0}M_{00}.$$

$I_{U}$ can generally be obtained, even in a laser polarimeter, through measurement prescriptions like those noted in Section 3.1 and Section 3.2.

The *reduced irradiance* of the ${\left(m\times n\right)}^{th}$ polarimeter measurement is then defined as

(12) $$I_{m\times n}^{\prime }\equiv I_{m\times n}-I_{U}=\sum_{i=1}^{15}W_{i}^{\left(m\times n\right)}{\bar{M}}_{i}.$$

The dyadic that generates the reduced irradiance $I^{\prime }$ is then

(13) $${\tilde{W}}^{\prime }=\left[\begin{array}{cccccccccc} \sigma_{1}s_{0} & \sigma_{2}s_{0} & \sigma_{3}s_{0} & \sigma_{0}s_{1} & \sigma_{1}s_{1} & \ldots & \sigma_{0}s_{3} & \sigma_{1}s_{3} & \sigma_{2}s_{3} & \sigma_{3}s_{3} \end{array}\right]$$

and the reduced vector Mueller matrix is

(14) $${\tilde{M}}^{\prime }=\left[\begin{array}{cccccccc} M_{01} & M_{02} & M_{03} & M_{10} & \ldots & M_{31} & M_{32} & M_{33} \end{array}\right].$$

Appendix B proves that the set of ${\tilde{M}}^{\prime }$ is a vector space, and is equivalent to $R^{15}$. The reduced-Mueller vector space is denoted $M^{\prime }.$ Note ${\tilde{W}}^{\prime }$ and ${\tilde{M}}^{\prime }$ are *not* normalized, because the set of normalized matrices is not closed under scalar multiplication. The reduced instrument vectors are also in $M^{\prime }$, and it has the inner product

(15) $$I_{m\times n}^{\prime }=\sum_{i=1}^{15}W_{i}^{\left(m\times n\right)}{\bar{M}}_{i}={\tilde{W}}^{\prime }\cdot {\tilde{M}}^{\prime }.$$

The inner-product space $M^{\prime }$ provides a completely linear description of the Mueller elements, enabling efficient specification of optimal pMMP channels, without use of a pseudoinverse, as well as diverse classifiers through which field data can be mapped at high speed on edge processors. The reduced Mueller matrices are physical to the extent that the parent, unreduced Mueller matrices are physical. Higher-order statistics than Equation (2) have been used to show that some matrices in the cone defined by Equation (3) are non-physical [25,26], and these matrices have been termed *pre-Mueller matrices* [27]. Non-physical matrices in the Mueller cone are avoided by our pMMP design technique, since the measured or modeled input/training Mueller matrices, as well as the solution instrument matrices, are physical by construction.

The inner product utilized for our pMMP design, Equation (15), is over $R^{15}$ rather than $R^{16},$ and is constrained to the Mueller cone.

## 3. Partial Mueller-Matrix Polarimeter Design

The general pMMP design problem is stated as follows: Given a target Mueller matrix $M_{T}$ and a polarimeter configuration defined by a parameter set $\Theta =\left\{\theta_{1},\theta_{2},\ldots ,\theta_{Q}\right\},$ determine the set Θ that maximizes representation of the target matrix in the set of M×N polarimeter measurements $\left\{I_{m\times n}\right\},$m=1,…,M and n=1,…N. The polarimeter configuration is described by the Stokes parameters $S_{G}^{\left(m\right)}$ and $S_{A}^{\left(n\right)}$ in Equations (4) and (5), which depend on the parameters Θ, which are often angular orientations but can be any combination of polarization-modulator parameters (see Section 3.1 and Section 3.2). In general $Q=MQ_{m}+NQ_{n}$ where $Q_{m}$ and $Q_{n}$ are the number of hardware parameters per PSG and PSA state, respectively. Hardware components that produce two (or more) states per parameter setting have $Q_{m,n}<1;$ for instance $Q_{n}=1/2$ for the PSA polarizing prism assumed in Section 3.1 and Section 3.2.

The raw polarimeter measurements are expressed by Equation (10), and by applying Equations (12)–(14) these can be converted into the reduced irradiances

(16) $$I_{m\times n}^{\prime }=\sum_{i=1}^{15}W_{i}^{\prime (m\times n)}{\bar{M}}_{i}^{\prime }={\tilde{W}}^{\prime }\cdot {\tilde{M}}^{\prime }.$$

**Figure 2:**
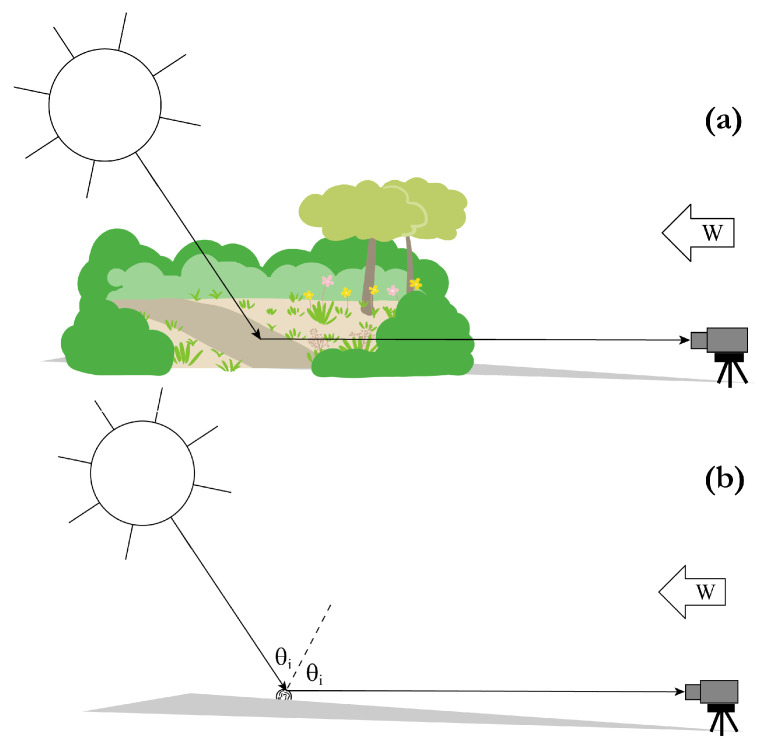
Schematic of an idealized passive remote sensing scenario for demonstration of the example 2-channel pMMP, measuring an arbitrary scene (**a**), which may or may not include the target, and measuring the target only (**b**). The target is a twisted dielectric cylinder running normal to the page. $\theta_{i}$ can obviously be converted to time of day.

Equation (16) expresses a polarimeter measurement of an arbitrary scene. A measurement of the target only is expressed as

(17) $$I_{Tm\times n}^{\prime }=\sum_{i=1}^{15}W_{i}^{\prime (m\times n)}{\bar{M}}_{Ti}^{\prime }={\tilde{W}}^{\prime }\cdot {\tilde{M}}_{T}^{\prime },$$

where ${\tilde{M}}_{T}^{\prime }$ is analogous to ${\tilde{M}}^{\prime }$ of Equation (14). Figure 2 depicts passive measurement scenarios corresponding to $I_{m\times n}^{\prime }$ and $I_{Tm\times n}^{\prime }.$ For remote sensing ${\tilde{M}}^{\prime }$ of Equation (16) must be arbitrary enough to account for any object that might be encountered, i.e., ${\tilde{M}}^{\prime }$ generally has 15 non-zero elements, but the target matrix ${\tilde{M}}_{T}^{\prime }$ is generally simplified based on a priori knowledge, i.e., ${\tilde{M}}_{T}^{\prime }$ has τ<15 non-zero elements. pMMP design can hence be interpreted as determination of the ${\tilde{W}}^{\prime }$ that maximizes representation of the τ non-zero elements of ${\tilde{M}}_{T}^{\prime }$ in the measurement $I_{T}^{\prime }.$

The target matrix ${\tilde{M}}_{T}^{\prime }$ is a τ-dimensional vector in the 15-dimensional vector space $M^{\prime }$ defined in Section 2. Moreover, the set of unit vectors in each of the τ dimensions of ${\tilde{M}}_{T}^{\prime },$${\left\{{\hat{M}}_{i}\right\}}_{i=1}^{\tau },$ forms the canonical basis of a τ-dimensional subspace (or hyperplane) of $M^{\prime },$ naturally denoted $M_{T}^{\prime }.$ The reduced target irradiance expressed by Equation (17) is therefore proportional to the cosine of the angle between the instrument vector ${\tilde{W}}^{\prime }$ and the target hyperplane $M_{T}^{\prime }$, while the arbitrary measurement of Equation (16) is proportional to the cosine between the same instrument vector and an arbitrary vector in $M^{\prime }.$ The projection of the instrument vector onto any of the 15−τ dimensions orthogonal to $M_{T}^{\prime }$ contributes no representation of the target signature to the polarimeter measurement, effectively wasting detector dynamic range on Mueller elements that provide no contrast and thereby lowering the overall contrast of the target against the rest of the scene. In order to maximize target contrast the instrument vector ${\tilde{W}}^{\prime }$ must be chosen to maximize its projection onto the target vector ${\tilde{M}}_{T}^{\prime }.$ Examples of ${\tilde{M}}_{T}^{\prime }$ are given in Section 3.1 and Section 3.2.

A gradient operator defined on $M^{\prime }$ as

(18) $$\nabla^{\prime }\equiv \sum_{i=1}^{15}{\hat{M}}_{i}^{\prime }\frac{\partial }{\partial {\bar{M}}_{i}^{\prime }}$$

and applied to measured irradiances, Equations (16) and (17), allows channels to be verified as linearly independent. As elaborated in Section 3.1 and Section 3.2, the M×N pMMP channels can be constrained by the requirement that the gradient vectors $\nabla^{\prime }I_{m\times n}^{\prime }$ be linearly independent. Those sections also show how the design technique can be applied to linear combinations of channel irradiances. As shown by example in Section 3.2, under-determined pMMPs typically offer several independent channels with identical maximum target irradiance $I_{Tm\times n}^{\prime }.$ These can often be further optimized in linear combinations, as in Section 3.2, or serve as inputs to classification algorithms, as discussed in Section 4.

Earlier pMMP design methods follow the demodulation approach used for complete Mueller-matrix polarimeters (MMPs), stacking irradiances of multiple channels, each a version of Equation (10), to form a *data-reduction matrix* (DRM) that is then inverted. But for typical under-determined pMMPs the DRM is not square, and this approach must either artificially reduce the dimension of the Mueller matrix, to obtain a smaller square DRM [23], or rely on pseudoinverses, which introduce computational instability or at least additional conditioning requirements [20,21]. The pMMP design method introduced here is not a modified DRM method; it optimizes each channel independently, affording computational simplicity and flexibility in channel definition. For instance, different channels of the same pMMP can be optimized to different objects that may occur in the same scene, and Section 4 shows channel flexibility by extension to a non-linear combination representative of the SBR statistic critical in remote sensing.

### 3.1. Two-Channel Passive Polarimeter

Passive optical polarimeters rely on solar illumination. Direct sunlight is typically unpolarized, while indirect sunlight may be partially linearly polarized due to atmospheric Rayleigh scattering. Direct solar illumination will be assumed here, implying the normalized illumination Stokes parameters

(19) $$S_{G}={\left[\begin{array}{cccc} 1 & 0 & 0 & 0 \end{array}\right]}^{T}.$$

For passive polarimeters m=M=1. A common PSA hardware configuration employs a polarizing prism, of which there are several types, to split the collected power into two signals or images with orthogonal polarization states, typically orthogonal linear states [28]. Here the polarizing prism is assumed to split the collected power into horizontal and vertical linear states. This configuration can be generalized, as assumed here, by placing a half-waveplate in front of the polarizing prism. If the waveplate crystal axis makes the angle $\theta_{r}$ with the horizontal axis, then the waveplate effectively rotates the polarizing prism by the angle $2\theta_{r}.$ Such a configuration is used in prominent astronomical telescopes, with a Wollaston prism projecting the orthogonal images to adjacent halves of a single digital camera [29]. Multiplication of the waveplate and prism Mueller matrices [10] specifies the PSA Stokes parameters for this configuration as

(20) $$S_{A}^{\left(n\right)}=\frac{1}{2}\left[\begin{array}{cccc} 1 & {\left(-1\right)}^{n}\cos\left(4\theta_{r}\right) & {\left(-1\right)}^{n}\sin\left(4\theta_{r}\right) & 0 \end{array}\right],$$

where n=1,2 specifies the prism output port. Applying Equations (19) and (20) to a general Mueller matrix per Equation (6), the general measured irradiance is given by

(21) $$I_{n}=\frac{1}{2}\left(M_{00}+{\left(-1\right)}^{n}M_{10}\cos\left(4\theta_{r}\right)+{\left(-1\right)}^{n}M_{20}\sin\left(4\theta_{r}\right)\right).$$

The unpolarized irradiance could be obtained by imaging an object with $M_{10}=M_{20}=0,$ although this is impractical in many field scenarios. More reliable is to form the difference of the two channels, in which the unpolarized irradiance is naturally eliminated,

(22) $$\Delta I^{\prime }=I_{2}-I_{1}=M_{10}\cos\left(4\theta_{r}\right)+M_{20}\sin\left(4\theta_{r}\right),$$

and the notation indicates that the difference is a reduced irradiance that can be optimized using the current method. Indeed, since Equations (16)–(18) are linear in the instrument matrix, the current pMMP design method can be applied to any linear combination of reduced irradiances; these may be referred to as “linear combinations of channels,” even though the channels must be individually implemented. Linear-combination channels require two or more hardware channels. Equation (22) can also be obtained by forming the reduced instrument matrix of this pMMP, from Equations (13), (19), and (20),

(23) $${\tilde{W}}_{n}^{\prime }=\frac{1}{2}\left[\begin{array}{ccccccccccccccc} 0 & 0 & 0 & {\left(-1\right)}^{n}\cos\left(4\theta_{r}\right) & 0 & 0 & 0 & {\left(-1\right)}^{n}\sin\left(4\theta_{r}\right) & 0 & 0 & 0 & 0 & 0 & 0 & 0 \end{array}\right],$$

and then using ${\tilde{W}}_{2}^{\prime }-{\tilde{W}}_{1}^{\prime }$ as the difference channel. Imaging of differential irradiance like that expressed by Equation (22) is commonly referred to as *polarization-difference imaging* (PDI) [30].

Figure 2 depicts a simple yet non-trivial example to illustrate PDI optimization. In Figure 2a the specified two-channel passive pMMP records or images an arbitrary scene, which may or may not include the target of interest. In Figure 2b the pMMP is oriented to record or image a cylindrical target of interest, for instance a fiberglass bundle, with its axis horizontal, for simplicity, perpendicular to the vertical plane containing both the sun and the polarimeter. Some strip of the cylinder is assumed to reflect sunlight specularly, to the polarimeter, for any solar angle $\theta_{i}$ in the vertical plane. The fibers are furthermore assumed to exhibit morphological diattenuation oriented at a fixed twist angle $\theta_{Di}$ relative to the cylinder axis. In this geometry the cylinder therefore exhibits diattenuation through two competing effects: fixed twist anisotropy and bistatic reflection, the latter varying with the solar angle and peaking at the Brewster angle $\theta_{i}=\theta_{B}$. In this example the pMMP will be optimized by expressing the differential irradiance of Equation (22) in terms of the object, illumination, and modulator parameters, and maximizing it.

The Mueller matrix of this idealized target is specified by multiplying the Mueller matrix of a linear diattenuator, with assumed 5% diattenuation at orientation $\theta_{Di}$ [10], by the Mueller matrix of a glass reflector at angle of incidence $\theta_{i}$ [31].

**Figure 3:**
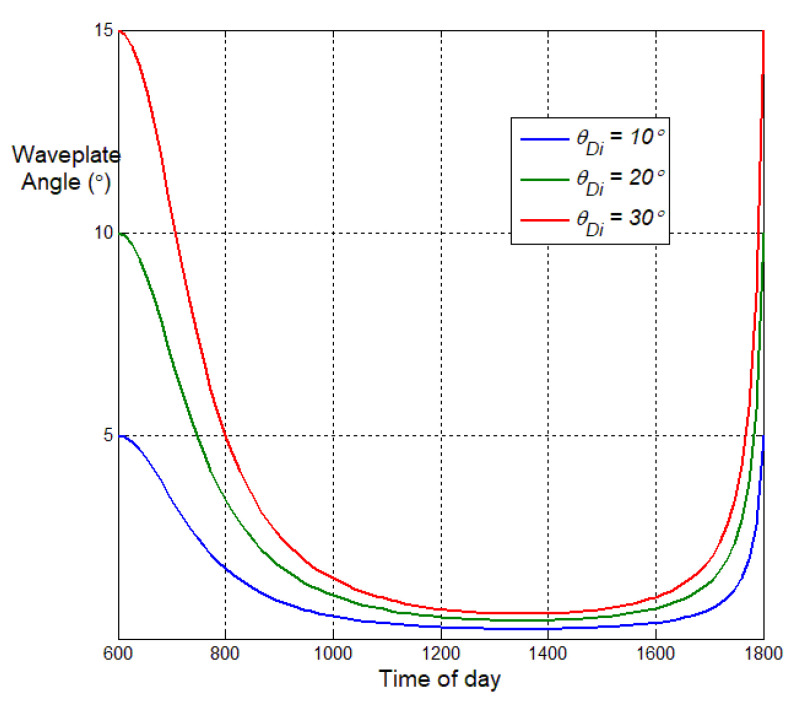
Optimal waveplate angle setting vs. time of day of 2-channel passive pMMP depicted in Figure 2, for detection of diattenuating cylinder in 3 twist angles.

The only two elements that affect the example pMMP are

(24) $$\begin{array}{ccc} M_{10} & = & \frac{1}{2}\left[\left(\rho_{⊥}-\rho_{∥}\right)+0.05\left(\rho_{⊥}+\rho_{∥}\right)\cos\left(2\theta_{Di}\right)\right]\\ & & \mathrm{and} \end{array}$$

(25) $$\begin{array}{ccc} M_{20} & = & 0.025\left(\rho_{⊥}+\rho_{∥}\right)\sin\left(2\theta_{Di}\right),\;\;\;\;\;\; \end{array}$$

where $\rho_{⊥}$ & $\rho_{∥}$ are complicated functions of $\theta_{i},$ defined in Ref. [31]. At normal incidence ($\theta_{i}=0$), $\rho_{⊥}=\rho_{∥}$ and only the twist anisotropy matters, while at the Brewster angle ($\theta_{i}=\theta_{B}$), $\rho_{∥}=0$ and the reflected light is mostly horizontally polarized. To maximize the projection ${\tilde{W}}^{\prime }\cdot {\tilde{M}}_{T}^{\prime },$ the derivative of Equation (22) is set to zero, resulting in

(26) $$\theta_{r\;\max}=\frac{1}{4}\tan^{-1}\left(\frac{M_{20}}{M_{10}}\right),$$

which is plotted in Figure 3 vs. solar angle and several values of $\theta_{Di}$. At sunrise (0600) the waveplate setting should be half the twist-anisotropy angle, but by late morning the optimal angle setting falls to near horizontal $\left(<1^{\circ }\right)$, where it stays for about 6h until rising again at sunset. This also shows that discrimination of the twist feature should be most feasible in the early morning, or early evening if the sensor is facing east.

### 3.2. Four-Channel Active Polarimeter

Active optical polarimeters typically employ laser illuminators. The illumination polarization state can be controlled, providing access to more details of the target signature/Mueller matrix than possible with passive polarimeters, also independent of weather and time of day. For demonstration, here the active polarimeter is assumed to comprise the same PSA specified in Section 3.1 for the passive polarimeter, i.e.,

(27) $$S_{A}^{\left(n\right)}=\frac{1}{2}\left[\begin{array}{cccc} 1 & {\left(-1\right)}^{n}\cos\left(4\theta_{r}\right) & {\left(-1\right)}^{n}\sin\left(4\theta_{r}\right) & 0 \end{array}\right],$$

and to transmit two PSG states, each generated by the ordered combination of a half-waveplate and a quarter-waveplate with crystal-axis orientations $\theta_{h}^{\left(m\right)}$ and $\theta_{q}^{\left(m\right)},$ respectively, with m=1,2. With a means of multiplexing the polarization states, which is generally not critical to the pMMP design, this configuration constitutes an M×N=4-channel active polarimeter. In this configuration the active channels are denoted by the angle triplet $\left(\theta_{h},\theta_{q},\theta_{r}\right).$ While this configuration is used for demonstration, the pMMP design method is general enough for any active-polarimeter hardware configuration with any number of measurement channels.

Assuming a vertical linearly polarized laser source, multiplying the half-waveplate and quarter-waveplate Mueller matrices [10] allows the PSG states to be expressed as

(28) $$\begin{array}{ccc} S_{G}^{\left(m\right)} & = & \left[\begin{array}{cc} 1 & -\cos^{2}\left(2\theta_{q}^{\left(m\right)}\right)\cos\left(4\theta_{h}^{\left(m\right)}\right)-\sin\left(4\theta_{q}^{\left(m\right)}\right)\sin\left(4\theta_{h}^{\left(m\right)}\right)/2 \end{array}\right.\\ & & \left.-\sin\left(4\theta_{q}^{\left(m\right)}\right)\cos\left(4\theta_{h}^{\left(m\right)}\right)/2-\sin^{2}\left(2\theta_{q}^{\left(m\right)}\right)\sin\left(4\theta_{h}^{\left(m\right)}\right)\right.\\ & & {\left.-\sin\left(2\theta_{q}^{\left(m\right)}\right)\cos\left(4\theta_{h}^{\left(m\right)}\right)+\cos\left(2\theta_{q}^{\left(m\right)}\right)\sin\left(4\theta_{h}^{\left(m\right)}\right)\right]}^{T}\\ & = & {\left[\begin{array}{cccc} 1 & g_{1}^{\left(m\right)} & g_{2}^{\left(m\right)} & g_{3}^{\left(m\right)} \end{array}\right]}^{T} \end{array}$$

with

(29) $$\begin{array}{ccc} g_{1}^{\left(m\right)} & \equiv & -\cos^{2}\left(2\theta_{q}^{\left(m\right)}\right)\cos\left(4\theta_{h}^{\left(m\right)}\right)-\sin\left(4\theta_{q}^{\left(m\right)}\right)\sin\left(4\theta_{h}^{\left(m\right)}\right)/2; \end{array}$$

(30) $$\begin{array}{ccc} g_{2}^{\left(m\right)} & \equiv & -\sin\left(4\theta_{q}^{\left(m\right)}\right)\cos\left(4\theta_{h}^{\left(m\right)}\right)/2-\sin^{2}\left(2\theta_{q}^{\left(m\right)}\right)\sin\left(4\theta_{h}^{\left(m\right)}\right); \end{array}$$

(31) $$\begin{array}{ccc} g_{3}^{\left(m\right)} & \equiv & -\sin\left(2\theta_{q}^{\left(m\right)}\right)\cos\left(4\theta_{h}^{\left(m\right)}\right)+\cos\left(2\theta_{q}^{\left(m\right)}\right)\sin\left(4\theta_{h}^{\left(m\right)}\right).\; \end{array}$$

Combining Equations (27) and (28) per Equation (6) expresses the general measured irradiance as

(32) $$\begin{array}{ccc} I_{m\times n} & = & \left[M_{00}+g_{1}^{\left(m\right)}M_{01}+g_{2}^{\left(m\right)}M_{02}+g_{3}^{\left(m\right)}M_{03}+{\left(-1\right)}^{n}M_{10}\cos\left(4\theta_{r}\right)+{\left(-1\right)}^{n}M_{11}g_{1}^{\left(m\right)}\cos\left(4\theta_{r}\right)\right.\\ & & +{\left(-1\right)}^{n}M_{12}g_{2}^{\left(m\right)}\cos\left(4\theta_{r}\right)+{\left(-1\right)}^{n}M_{13}g_{3}^{\left(m\right)}\cos\left(4\theta_{r}\right)+{\left(-1\right)}^{n}M_{20}\sin\left(4\theta_{r}\right)\\ & & \left.+{\left(-1\right)}^{n}M_{21}g_{1}^{\left(m\right)}\sin\left(4\theta_{r}\right)+{\left(-1\right)}^{n}M_{22}g_{2}^{\left(m\right)}\sin\left(4\theta_{r}\right)+{\left(-1\right)}^{n}M_{23}g_{3}^{\left(m\right)}\sin\left(4\theta_{r}\right)\right]. \end{array}$$

This pMMP design can measure most Mueller elements and features. The active polarimeter can measure the unpolarized irradiance $M_{00}$ directly, specifically by summing channel pairs $I_{m\times 1}$ and $I_{m\times 2}$ with PSG states satisfying

(33) $$\sum_{m}g_{1}^{\left(m\right)}=\sum_{m}g_{2}^{\left(m\right)}=\sum_{m}g_{3}^{\left(m\right)}=0;$$

$M_{00}$ so obtained can then be subtracted from other measured channels to form the reduced irradiance per Equation (12). Alternatively, $M_{00}$ can be eliminated, as in the passive polarimeter, by differencing each pair of measured irradiances $I_{m\times 1}$ and $I_{m\times 2}$. For simplicity this approach is adopted below.

Further assuming the reciprocity conditions of Equations (A16) and (A17), the reduced measured irradiance in the $m^{th}$ difference channel is expressed as

(34) $$\begin{array}{ccc} \Delta I_{Rm}^{\prime } & \equiv & I_{Rm\times 2}^{\prime }-I_{Rm\times 1}^{\prime }\\ & = & M_{01}\cos\left(4\theta_{r}\right)-M_{02}\sin\left(4\theta_{r}\right)+M_{11}g_{1}^{\left(m\right)}\cos\left(4\theta_{r}\right)\\ & & +M_{12}\left[g_{2}^{\left(m\right)}\cos\left(4\theta_{r}\right)-g_{1}^{\left(m\right)}\sin\left(4\theta_{r}\right)\right]+M_{13}g_{3}^{\left(m\right)}\cos\left(4\theta_{r}\right)\\ & & +M_{22}g_{2}^{\left(m\right)}\sin\left(4\theta_{r}\right)+M_{23}g_{3}^{\left(m\right)}\sin\left(4\theta_{r}\right). \end{array}$$

The active pMMP provides multiple difference channels, compared to the single difference channel of the passive pMMP, each of which can be optimized per the design method once a target Mueller matrix is defined. The active channels are typically further constrained to have linearly independent gradient vectors, obtained by applying Equation (18) to Equation (34). Measured data from these channels can be passed to a machine learning algorithm for generalized classification derived from a training dataset obtained from any model or measurement of the Mueller elements that appear in the pMMP channels. Training processes may generally include background and clutter polarization signatures in addition to the target signature(s), the incorporation of which is considered in Section 4, where signal-to-background ratio (SBR) optimization is accommodated.

Next an active target must be defined. A common application for a laser sensor is classification of man-made objects on and within natural backgrounds. Furthermore, laser sensors are usually monostatic or quasi-monostatic. In this scenario the target Mueller matrix is sometimes taken as that of an ideal partial depolarizer [32], i.e.,

(35) $$M_{T}=\mathrm{diag}\left(1,\alpha ,\alpha ,\beta \right),$$

with α≈β≈1 for a smooth metal. Assuming this target, substituting into Equation (34) gives the reduced target irradiance in the difference channel as

(36) $$\Delta I_{Tm}^{\prime }=\alpha \left(g_{1}^{\left(m\right)}\cos\left(4\theta_{r}\right)+g_{2}^{\left(m\right)}\sin\left(4\theta_{r}\right)\right).$$

Computing this differential irradiance over the 3D array $\left(\theta_{h},\theta_{q},\theta_{r}\right)$ reveals it is maximized for $\left(\theta_{h},\theta_{q},\theta_{r}\right)=\left(\theta_{q}/2,\theta_{q},\theta_{q}/2\right),$ for which $g_{1}=-\cos\left(2\theta_{q}\right),$ $g_{2}=-\sin\left(2\theta_{q}\right),$ $g_{3}=0,$ and $\left|\Delta I_{T}^{\prime }\right|=\alpha .$ For these channels, the general reduced irradiance (Equation (34) of a reciprocal scene becomes

(37) $$\Delta I_{Rm}^{\prime }=M_{01}\cos\left(2\theta_{q}^{\left(m\right)}\right)-M_{02}\sin\left(2\theta_{q}^{\left(m\right)}\right)-M_{11}\cos^{2}\left(2\theta_{q}^{\left(m\right)}\right)-M_{22}\sin^{2}\left(2\theta_{q}^{\left(m\right)}\right),$$

with corresponding gradient vector

(38) $$\nabla^{\prime }\Delta I_{Rm}^{\prime }={\hat{M}}_{01}\cos\left(2\theta_{q}^{\left(m\right)}\right)-{\hat{M}}_{02}\sin\left(2\theta_{q}^{\left(m\right)}\right)-{\hat{M}}_{11}\cos^{2}\left(2\theta_{q}^{\left(m\right)}\right)-{\hat{M}}_{22}\sin^{2}\left(2\theta_{q}^{\left(m\right)}\right).$$

Table 1 lists pMMP solution channels that can be further combined to enhance representation of this target. Channels 1–4 are linearly independent; channels 5–6 are linear combinations of channels 1–4 that help elucidate the design criteria discussed below.

The optimal difference channels in Table 1 still have components orthogonal to the target hyperplane $M_{T}^{\prime }$. For example, from Equation (38), $\nabla^{\prime }\left(\Delta I_{R1}^{\prime }\right)={\hat{M}}_{01}-{\hat{M}}_{11}$ and $\nabla^{\prime }\left(\Delta I_{R3}^{\prime }\right)=-{\hat{M}}_{01}-{\hat{M}}_{11}.$ The orthogonal components can be completely eliminated by forming linear combinations of the difference channels, in this case sums of differences, like $\Sigma \Delta I_{R1}^{\prime }\equiv \Delta I_{R1}^{\prime }+\Delta I_{R3}^{\prime }=-2M_{11}$ from Equation (37), for which $\left|\Sigma \Delta I_{T}^{\prime }\right|=2\alpha ,$ demonstrating the advantage of the 4-channel configuration. Unlike channels 1–4, channels 5 and 6 each represent *two* target Mueller elements ($M_{11}$ and $M_{22}$), which could be advantageous in many scenarios, but at the expense of retaining two orthogonal components ($M_{01}$ and $M_{02}$), e.g., $\nabla^{\prime }\left(\Delta I_{R5}^{\prime }\right)=\frac{1}{\sqrt{2}}\left[{\hat{M}}_{01}-{\hat{M}}_{02}-\left({\hat{M}}_{11}+{\hat{M}}_{22}\right)/\sqrt{2}\right].$ The orthogonal components effectively lower the target representation in the pMMP channel. Channels 5–6 can likewise be combined to eliminate orthogonal components, i.e., $\nabla^{\prime }\left(\Sigma \Delta I_{R5}^{\prime }\right)=\nabla^{\prime }\left(\Delta I_{R5}^{\prime }+\Delta I_{R6}^{\prime }\right)=-{\hat{M}}_{11}-{\hat{M}}_{22}$. These results can be interpreted in the context of the bias-variance tradeoff discussed in Section 4.

The channels specified in Table 1 are optimal for the ideal partial depolarizer represented by Equation (35). A great variety of other objects and materials of interest in remote sensing and microscopy exhibit Mueller matrices with off-diagonal elements, for which the optimal channels are obviously different. Certain materials of interest, for instance plastics [13] and atmospheric aerosols [33], exhibit retardance ($M_{13}$ and $M_{23}$) that converts a fraction of linear polarization to circular polarization. Referring to Equations (29)–(31) and (34), the example four-channel active polarimeter can be tuned to maximize the differential irradiance of such targets by tuning the waveplate angles such that $g_{3}\neq 0.$ In biomedical optics, use of elliptical polarization has been shown to enable 3D imaging of layered biological tissues and fluids [4]. The example active pMMP can be constrained to provide elliptical illumination by setting $0<\left|g_{3}\right|\leq 1$ (maximum ∼ circular). The corresponding differential irradiance of a target (Equation (34) for a reciprocal target) could then be maximized, and linear-combination channels could be formed to eliminate non-target projections, which would include $g_{3}$ components in Equation (34) for targets without retardance.

## 4. Classifier Design

The *bias–variance tradeoff* is a cornerstone of machine learning that balances performance on training datasets with the ability to generalize to unforeseen data. For automated- or aided-target-recognition (ATR) algorithms or classifiers, the smallest possible training dataset comprises the target only. Excluding background and clutter signatures from the training dataset results in a highly biased classifier. Likewise, relying on a single channel or feature lowers the classifier dimension, limiting the amount of variance that can be introduced. Referring to the example of Section 3.2, the pMMP design method indicates that the sum-difference channels $\Sigma \Delta I_{R1}^{\prime },\Sigma \Delta I_{R3}^{\prime },$ and $\Sigma \Delta I_{R5}^{\prime }$ are all parallel to the target subspace of the partial depolarizer (Equation (35)), but $\Sigma \Delta I_{R5}^{\prime }$ has more variance since it spans both dimensions ${\hat{M}}_{11}$ and ${\hat{M}}_{22}$ in $M_{T}^{\prime }$. For instance, if the target matrix is generalized to include noise, such that $M_{T\delta }=$ diag$(1,\alpha +\delta_{1},\alpha +\delta_{2},\beta +\delta_{3}),$ where $\delta_{i}$ are small real random variables, then channel $\Sigma \Delta I_{R5}^{\prime }$ will outperform channels $\Sigma \Delta I_{R1}^{\prime }$ and $\Sigma \Delta I_{R3}^{\prime }$ when applied to such an ensemble of targets.

There are several approaches to increase the classifier variance. The pMMP design method itself can be extended to incorporate background and clutter signatures, for instance by minimizing projections onto the corresponding Mueller matrices. If the background material is characterized by the Mueller matrix $M_{b}$ and the reduced irradiance $I_{bm\times n}^{\prime },$ then a signal-to-background ratio (SBR) can be defined as

(39) $$R_{m\times n}\equiv \frac{I_{Tm\times n}^{\prime }}{I_{bm\times n}^{\prime }}.$$

SBR presents a non-linear channel combination that the new pMMP design technique can uniquely accommodate, for instance by maximizing the ratio for each channel as

(40) $$\max R_{m\times n}=\max_{\Theta }\left[\frac{I_{Tm\times n}^{\prime }}{I_{bm\times n}^{\prime }}\right].$$

Alternatively or in combination, data acquired in the optimized pMMP channels can be used to train a machine learning algorithm, a support-vector machine (SVM) [16,17,18,34] or a myriad of others, at which stage techniques like regularization and penalty functions can be introduced to increase the variance.

Since there are many options for classifier generalization, it is useful to define a homogeneous maximally-biased classifer for a pMMP design, to serve as a baseline by which to compare generalized classifiers. The pMMP design method allows all measured linearly independent pMMP channels to be combined into a single linear-combination channel applicable as a maximally-biased classifier with bias homogenously distributed among the channels. Returning to the active polarimeter of Section 3.2, per Equation (37) the reduced measured irradiance in the combined difference channel is defined as

(41) $$\Delta I_{R\Sigma }^{\prime }\equiv A\Delta I_{R1}^{\prime }+B\Delta I_{R2}^{\prime }+C\Delta I_{R3}^{\prime }+D\Delta I_{R4}^{\prime },$$

and the combined channel can be optimized by maximizing this differential irradiance, now for an eight-channel pMMP. In addition to providing a baseline for classifier generalization, the maximally biased classifier allows the polarimeter to be demonstrated and applied prior to potentially costly algorithm training and without the processing overhead needed to apply more generalized algorithms in real time.

Classifiers derived from the new pMMP technique can be conceptualized by comparison with other classifiers that have utilized Mueller matrices. Most prior polarimetric classifiers are designed to discriminate between a target and a background Mueller matrix or distributions thereof [34,35,36]. These algorithms implicitly assume that the magnitude of the background irradiance is similar to that of the target, or at least of SNR adequate to quantify separation from the target irradiance. On the contrary, the pMMP design technique introduced here preferably derives channels in which the background irradiance vanishes, according to Equation (40) for instance. This is more consistent with the common PDI notion to select channels in which the background irradiance is identical, and so vanishes in the difference channel, while the target irradiance is different in the raw channels, and so survives differencing. Two or more pMMP channels derived according to this criterion, Equation (40) for instance, can then be combined in a classifier designed to maximize separation, for instance between the target and one or more clutter signatures.

Because this pMMP technique operates on reduced Mueller matrices, it cannot be applied directly to distinguish among objects or materials represented by the same reduced Mueller matrices but different depolarization indices [37]. For instance, if a target represented by Equation (35) is on or within a background represented by $M_{b}=$ diag(2,α,α,β), then the derived channels (eg. those derived in Section 3.2) will still maximize the reduced (e.g., differential) irradiance of the target, but will not separate the target from the background. In such scenarios the pMMP can be extended to utilize the full irradiance (Equation (10)) and the classifier can be augmented to employ depolarization index [32].

## 5. Discussion

Application-specific sensors should be justifiable considering the vast amounts of time and cost spent (often wasted) trying to adapt general-purpose sensors to remote sensing tasks. “Sensors of opportunity” rarely perform well, and can even discourage further development of sensor modalities that could be well-suited to the task if optimally designed. This paper presents a general technique to do so for polarization sensors.

While the polarimeter architectures assumed in Section 3.1 and Section 3.2 are for optical frequencies, the pMMP design technique is applicable to any frequency of electromagnetic waves, in particular radio frequencies, with specific prescriptions for the associated hardware, modulators, and signatures. It should also be clear, from the generality of this treatment, that any measurement of linear polarization properties of any object can be expressed as a pMMP, and therefore optimized using this technique.

Mathematically, the reduced-Mueller vector space and inner-product space introduced here enable a completely linear pMMP analysis that is still constrained to the Mueller cone. This space provides a basis to describe the geometrical behavior of Mueller matrices via sets of matrices with the same reduced-Mueller vector. As one implication, the geometrical description and depiction of non-physical matrices in the Mueller cone, and how these might affect the pMMP design technique, is a topic for future research.

## Figures and Tables

**Table 1:** Parameters and channel designations for the example 4-channel active pMMP.

*m* =	1	2	3	4	5	6
$\theta_{q}\left({}^{\circ}\right)$	0	45	90	135	22.5	−67.5
$\theta_{h}\left({}^{\circ}\right)$	0	22.5	45	67.5	11.25	−33.75
$\theta_{r}\left({}^{\circ}\right)$	0	22.5	45	67.5	11.25	−33.75
$g_{1}$	−1	0	1	0	$-1/\sqrt{2}$	$1/\sqrt{2}$
$g_{2}$	0	−1	0	1	$-1/\sqrt{2}$	$1/\sqrt{2}$
$g_{3}$	0	0	0	0	0	0
$\left|\Delta I_{T}^{\prime }\right|$	α	α	α	α	α	α

## Data Availability

No new data was created for this work.

## Abbreviations

The following abbreviations are used in this manuscript:

ATR	Automated or aided target recognition
DoA	Division of amplitude
DoT	Division of time
DRM	Data-reduction matrix
MMP	Mueller-matrix polarimeter
PDI	Polarization-difference imaging
PSA	Polarization-state analyzer
PSG	Polarization-state generator
SBR	Signal-to-background ratio
SNR	Signal-to-noise ratio
SVM	Support-vector machine

## Appendix A. Relevant Properties of the Mueller Cone

### Appendix A.1. Definition of a Cone

Let $\left(R^{n}.∥-∥\right)$ be a normed real vector space of finite dimension. A cone $K\subset R^{n}$ is a set closed under nonnegative linear combinations and limits, and the only vector in *K* whose additive inverse is also in *K* is the zero vector. That is, *K* is a cone if and only if

1. For all v,w∈K and α,β≥0, αv+βw∈K.

2. For all ${\left\{v_{n}\right\}}_{n\in N}\subseteq K$ such that $\lim_{n\to \infty }v_{n}=\hat{v},$ then $\hat{v}\in K.$

This means *K* is a closed set under the topology given by the norm.

3. The set $K\cap \left(-K\right)=\left\{\bar{0}\right\}$ where $-K=\{-v\in R^{n}$|v∈K}.

The topological basis is expressed in terms of open balls of radius *r* centered at x, that is

(A1) $$B_{r}\left(x\right)=\left\{y\in R^{n}|∥x-y∥<r\geq 0\right\},$$

and the cone is *solid* if it has a non-empty interior, that is

(A2) $$int_{R^{n}}\left(K\right)=\left\{x\in K\;|\;\exists r>0,B_{r}\left(x\right)\subset K\right\}.$$

### Appendix A.2. Examples

The set of nonnegative vectors of $R^{n}$, denoted by $R_{+}^{n}$ or ${\left(R^{n}\right)}^{+}$, is a solid cone with the usual norm

(A3) $$∥[\begin{array}{ccc} v_{1} & \ldots & v_{n} \end{array}]∥\;=\sqrt{v_{1}^{2}+\ldots +v_{n}^{2}}.$$

This cone is called the positive cone or the nonnegative cone of $R^{n}$.

The set of the Stokes vectors in $R^{4}$ forms a solid cone with the usual norm. Namely,

(A4) $$\begin{array}{ccc} K & = & \left\{{\left[\begin{array}{cccc} a & x & y & z \end{array}\right]}^{T}\in R^{4}\;|\;a\geq 0\;\&\;a^{2}\geq x^{2}+y^{2}+z^{2}\right\}\\ & = & \{S={\left[\begin{array}{cc} a & v \end{array}\right]}^{T}\in R^{4}\;|\;a\geq 0\;\&\;v\in R^{3}\;\&\;S^{T}\mathrm{GS}\geq 0\}, \end{array}$$

where $S^{T}$ denotes the transpose of S and G= diag(1,−1,−1,−1). Vectors for which $S^{T}\mathrm{GS}=0,$ which represent fully-polarized states, form the boundary of the cone, ensuring *K* is topologically closed. Vectors with $S^{T}\mathrm{GS}>0,$ partially-polarized states, are in the interior of K.

Mueller matrices M map Stokes vectors to Stokes vectors, according to Equation (1). In other words, M leaves the Stokes cone invariant, i.e., M(K)⊆K. Combining Equation (A4) with Equation (1), Mueller matrices must satisfy

(A5) $$S^{T}M^{T}\mathrm{GMS}\geq 0$$

for all S. Convex sets of Mueller matrices are known to combine to form new Mueller matrices, and any physical Mueller matrix can be expressed as a convex sum of so-called *pure* Mueller matrices [9] or *Jones–Mueller matrices* [38]. Mueller matrices therefore satisfy condition 1 above through their convex sums. It is also well-known that Mueller matrices, like Stokes vectors, lack additive inverses, and so satisfy condition 3 above. To show that the set of Mueller matrices satisfies condition 2, let ${\left\{M^{\left(n\right)}\right\}}_{n\in N}$ be a collection of Mueller matrices whose limit is the matrix $\hat{M}$: $\lim_{n\to \infty }M^{\left(n\right)}=\hat{M}$. If S is a Stokes vector, then $M^{\left(n\right)}S\in K.$$\lim_{n\to \infty }M^{\left(n\right)}S=\hat{M}S$ is a limit of the Stokes vectors, which must also be in the Stokes cone since it is topologically closed, i.e., $\hat{M}S\in K.$ The set of Mueller matrices is therefore topologically closed, and the set of Mueller matrices therefore forms the cone

(A6) $$\tilde{K}=\left\{M\in R^{4\times 4}|\;M\left(K\right)\subseteq K\right\}.$$

It is also clear that the vectorized form of M, $\tilde{M}$ from Equation (), forms an isomorphic cone.

For example, the Jones-Mueller matrices $M_{J}$ map Stokes vectors from the boundary to the boundary of the Stokes cone, such that $S^{T}M_{J}^{T}{\mathrm{GM}}_{J}S=0$ if $S^{T}\mathrm{GS}=0$ [38], and therefore belong to the boundary of the Mueller cone $\tilde{K}$.

## Appendix B. Proof of Reduced-Mueller Vector Space

**Proof.** For this proof define the 4×4 real matrices

(A7) $$E_{ij}\left(p,q\right)=\left\{\begin{array}{cc} 1 & \mathrm{if}\;(p,q)=(i,j)\\ 0 & \mathrm{otherwise} \end{array}\right.,$$

for all 0≤i,j≤3 [24]. For instance,

(A8) $$E_{00}=\left[\begin{array}{cccc} 1 & 0 & 0 & 0\\ 0 & 0 & 0 & 0\\ 0 & 0 & 0 & 0\\ 0 & 0 & 0 & 0 \end{array}\right],$$

the Mueller matrix of the ideal depolarizer, is in the interior of $\tilde{K}.$ Condition (A5) can be applied to show that $E_{00}+E_{ij}$ and $E_{00}-E_{ij}$ are in $\tilde{K}$ for all 0≤i,j≤3. The set of reduced Mueller matrices is denoted by

$$\begin{array}{c} M^{\prime }=\left\{{\tilde{M}}^{\prime }=\left[\begin{array}{cccccccc} M_{01} & M_{02} & M_{03} & M_{10} & \ldots & M_{31} & M_{32} & M_{33} \end{array}\right]\in R^{15}|\right. \end{array}$$

(A9) $$\begin{array}{c} \left.\tilde{M}=\left[\begin{array}{ccccccccc} M_{00} & M_{01} & M_{02} & M_{03} & M_{10} & \ldots & M_{31} & M_{32} & M_{33} \end{array}\right]\in \tilde{K}\right\}. \end{array}$$

That is, each Mueller matrix is recast as its line-vector in $R^{16},$ and $M_{00}$ is removed, resulting in a vector in $R^{15}$. Observe that for all $\tilde{M},\tilde{N}\in \tilde{K}$ and for all α,β≥0,$\alpha \tilde{M}+\beta \tilde{N}\in \tilde{K}$ and

(A10) $${\left(\alpha \tilde{M}+\beta \tilde{N}\right)}^{\prime }=\alpha {\tilde{M}}^{\prime }+\beta {\tilde{N}}^{\prime }.$$

Note that ${\left(E_{00}-E_{ij}\right)}^{\prime }=-{\left(E_{00}+E_{ij}\right)}^{\prime }$ is a reduced Mueller matrix. Let $v=\left[\begin{array}{ccc} v_{1} & \ldots & v_{15} \end{array}\right]\in R^{15}$ be *any* vector in $R^{15}.$ Any vector in $R^{15}$ is expressible as

(A11) $$v=\frac{1}{2}\left[\sum_{i,j=0}^{3}\left(1+\mathrm{sgn}(v_{4i+j})\right)v_{4i+j}{\left(E_{00}+E_{ij}\right)}^{\prime }+\sum_{i,j=0}^{3}\left(1-\mathrm{sgn}(v_{4i+j})\right)\left|v_{4i+j}\right|{\left(E_{00}-E_{ij}\right)}^{\prime }\right],$$

where

(A12) $$\mathrm{sgn}\left(\alpha \right)\equiv \left\{\begin{array}{c} 1\;\mathrm{if}\;\alpha >0\\ 0\;\mathrm{if}\;\alpha =0\\ -1\;\mathrm{if}\;\alpha \;<0 \end{array}\right..$$

Then, by Equation (A10),

(A13) $$v=\frac{1}{2}{\left[\sum_{i,j=0}^{3}\left(1+\mathrm{sgn}(v_{4i+j})\right)v_{4i+j}\left(E_{00}+E_{ij}\right)+\sum_{i,j=0}^{3}\left(1-\mathrm{sgn}(v_{4i+j})\right)\left|v_{4i+j}\right|\left(E_{00}-E_{ij}\right)\right]}^{\prime }.$$

But $\left[\sum_{i,j=0}^{3}\left(1+\mathrm{sgn}(v_{4i+j})\right)v_{4i+j}\left(E_{00}+E_{ij}\right)+\sum_{i,j=0}^{3}\left(1-\mathrm{sgn}(v_{4i+j})\right)\left|v_{4i+j}\right|\left(E_{00}-E_{ij}\right)\right]\in \tilde{K}$ because $\left(E_{00}+E_{ij}\right),\left(E_{00}-E_{ij}\right)\in \tilde{K}$ and $\left(1+\mathrm{sgn}(v_{4i+j})\right)v_{4i+j},\left(1-\mathrm{sgn}(v_{4i+j})\right)\left|v_{4i+j}\right|\geq 0.$ Therefore, every vector $v\in R^{15}$ is equal to a reduced Mueller matrix, and $M^{\prime }$ is equal to $R^{15}.$ □

## Appendix C. Symmetric Mueller Matrices

Symmetry in the Mueller matrix lowers the dimensionality of the pMMP design problem. Nearly all objects and media that might be encountered in remote sensing exhibit *reciprocity* in their Mueller matrices, i.e.,

(A14) $$M_{R}=\left[\begin{array}{cccc} M_{00} & M_{01} & M_{02} & M_{03}\\ M_{01} & M_{11} & M_{12} & M_{13}\\ -M_{02} & -M_{12} & M_{22} & M_{23}\\ M_{03} & M_{13} & -M_{23} & M_{33} \end{array}\right]$$

in reflection, implying 10 independent elements. Switching to the reduced Mueller matrix defined in Section 2 and expanding the expression for measured irradiance, Equation (16), with a reciprocal Mueller matrix reveals that

(A15) $$I_{Rm\times n}^{\prime }=\sum_{i=1}^{9}W_{Ri}^{\prime (m\times n)}M_{Ri}^{\prime }$$

where

(A16) $$\begin{array}{ccc} {\tilde{W}}_{R}^{\prime } & \equiv & \left[\begin{array}{ccccccc} \left(W_{1}^{\prime }+W_{4}^{\prime }\right) & \left(W_{2}^{\prime }-W_{8}^{\prime }\right) & \left(W_{3}^{\prime }+W_{12}^{\prime }\right) & W_{5}^{\prime } & \left(W_{6}^{\prime }-W_{9}^{\prime }\right) & \left(W_{7}^{\prime }+W_{13}^{\prime }\right) & W_{10}^{\prime } \end{array}\right.\\ & & \left.\begin{array}{cc} \left(W_{11}^{\prime }-W_{14}^{\prime }\right) & W_{15}^{\prime } \end{array}\right] \end{array}$$

and

(A17) $${\tilde{M}}_{R}^{\prime }\equiv \left[\begin{array}{ccccccccc} M_{01} & M_{02} & M_{03} & M_{11} & M_{12} & M_{13} & M_{22} & M_{23} & M_{33} \end{array}\right].$$

That is, reciprocal symmetry reduces the problem from 15 to 9 dimensions. The nine-dimensional vector space spanned by the unit vectors associated with ${\tilde{M}}_{R}^{\prime }$ is denoted $M_{R}^{\prime }.$ The reciprocity results of Equations (A15)–(A17) are utilized in Section 3.2.
